# Fully automated synthesis of (phospho)peptide arrays in microtiter plate wells provides efficient access to protein tyrosine kinase characterization

**DOI:** 10.1186/1471-2172-6-1

**Published:** 2005-01-12

**Authors:** Carl Saxinger, Thomas P Conrads, David J Goldstein, Timothy D Veenstra

**Affiliations:** 1 Center for Cancer Research, NCI, Building 1052, Frederick, MD, 21702, USA; 2 Laboratory of Proteomics and Analytical Technologies, SAIC-Frederick Inc., National Cancer Institute at Frederick, PO Box B, Frederick, MD, 21702, USA; 3 Center for Cancer Research, NCI, Building 31, Bethesda, MD 20892, USA

## Abstract

**Background:**

Synthetic peptides have played a useful role in studies of protein kinase substrates and interaction domains. Synthetic peptide arrays and libraries, in particular, have accelerated the process. Several factors have hindered or limited the applicability of various techniques, such as the need for deconvolution of combinatorial libraries, the inability or impracticality of achieving full automation using two-dimensional or pin solid phases, the lack of convenient interfacing with standard analytical platforms, or the difficulty of compartmentalization of a planar surface when contact between assay components needs to be avoided. This paper describes a process for synthesis of peptides and phosphopeptides on microtiter plate wells that overcomes previous limitations and demonstrates utility in determination of the epitope of an autophosphorylation site phospho-motif antibody and utility in substrate utilization assays of the protein tyrosine kinase, p60^c-src^.

**Results:**

The overall reproducibility of phospho-peptide synthesis and multiplexed EGF receptor (EGFR) autophosphorylation site (pY1173) antibody ELISA (9H2) was within 5.5 to 8.0%. Mass spectrometric analyses of the released (phospho)peptides showed homogeneous peaks of the expected molecular weights. An overlapping peptide array of the complete EGFR cytoplasmic sequence revealed a high redundancy of 9H2 reactive sites. The eight reactive phospopeptides were structurally related and interestingly, the most conserved antibody reactive peptide motif coincided with a subset of other known EGFR autophosphorylation and SH2 binding motifs and an EGFR optimal substrate motif. Finally, peptides based on known substrate specificities of c-src and related enzymes were synthesized in microtiter plate array format and were phosphorylated by c-Src with the predicted specificities. The level of phosphorylation was proportional to c-Src concentration with sensitivities below 0.1 Units of enzyme.

**Conclusions:**

The ability of this method to interface with various robotics and instrumentation is highly flexible since the microtiter plate is an industry standard. It is highly scalable by increasing the surface area within the well or the number of wells and does not require specialized robotics. The microtiter plate array system is well suited to the study of protein kinase substrates, antigens, binding molecules, and inhibitors since these all can be quantitatively studied at a single uniform, reproducible interface.

## Background

Phosphorylation and dephosphorylation of proteins are major mechanisms mediating signal transduction throughout the cell and are intimately involved in the regulation of cell growth, physiology, differentiation, and death. Phosphorylation is accomplished by means of kinases which when stimulated by an afferent signal transmit the signal via phosphate transfer to the next site in a pathway. In some cases phosphoprotein-protein interactions take place that modulate signal transduction, e.g. by revealing previously sequestered phosphoacceptor sites in one or both of the interacting proteins, thus creating branch points in pathways. Critical questions exist regarding the identification of the *true in vivo *substrates of kinases, identification of phosphotyrosine interaction domains, and mapping the radiation of these protein interactions throughout extremely complex networks. Clearly new technologies capable of accelerating the processes for defining the interactions between kinases and their substrates and modulators would be of great value.

Two highly productive approaches have been the determination of optimal substrate motifs favored by individual kinases, by various combinatorial peptide library approaches and, the use of antibodies to study phosphorylated peptide motifs (reviewed in [1,2]). Synthetic peptides have played a long and useful role in characterizing kinase substrate sequences, particularly for the ser/thr family, which is now seen to consist of a few distinct category types, basophilic, acidophilic and proline directed. Protein tyrosine kinases, on the other hand, are less well defined by their natural substrates but make more use of docking intermediaries to perform the task of substrate recognition. Nevertheless, optimal substrates have been found which can then aid in the search for the identity of natural or *in vivo *targets and inhibitors of the kinase [3,4]. While capable of assessing mixtures of very large numbers of random peptides, combinatorial methods require deconvolution strategies, which can be time-consuming, and technically demanding.

A second search strategy for functional peptides employs arrays of spatially addressable peptides that can be tested *in situ*, accelerating the deconvolution process when the number of combinations is, or becomes more limited. Peptide arrays capable of displaying diverse functions including kinase substrate activity have been successfully produced by two methods: *in situ *synthesis on planar membranes or arrays of pins [5-10], or attachment of preformed peptides as performed in a variety of microarray printing procedures. While the existing synthetic methods are capable of producing large numbers of peptides in good purity, none are fully automatable. They require manual intervention between each synthesis cycle and thus are not totally automatable. Peptide synthesis in microtiter plate wells would allow the use of fully automated robotic handlers. Further, arrays of peptides produced in a microtiter plate format, which is an industry standard for numerous types of high throughput analytical procedures, could also be tested in automated multiplex fashion.

We present data here demonstrating the applicability of an automated system for peptide design and synthesis in microtiter plates to the production of peptides and phosphopeptides. We further demonstrate the capability of these peptide arrays to be recognized correctly by specific phospho-motif antisera and to serve as kinase substrates.

## Results and discussion

### (Phospho)peptide synthesis method

**Figure 1:**
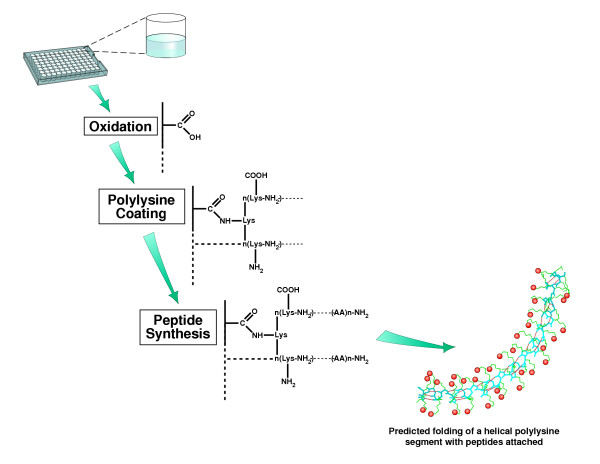
**Microtiter plate peptide array chemistry **Costar TPX microtiter plate strip well surfaces were oxidized with nitric acid, coated with polylysine and peptide synthesis is performed as described in Methods. Attachment of the polylysine chains to the wells and initiation of peptide synthesis takes place through the lysine ε-amino groups. The predicted polylysine helical segment shown is stabilized primarily through backbone H-bonding and synthesized peptides are depicted by red spheres.

Previous work led to the development of a system for the automated synthesis of peptide arrays on the inner surfaces microtiter plate wells [11-13], (Figure 1). However, direct characterization of the synthesized peptides did not become realizable until the recent availability of modern high-sensitivity mass spectrometers. In this method polymethylpentene (TPX) microtiter plates are activated by oxidation with nitric acid, and made functional for peptide synthesis by condensation with poly(D-lysine) (n = 100), which serves as a free-floating support or polymeric handle containing an extended array of amino groups. Each lysine side-chain then serves as an initiation point for synthesis. The synthesized peptides are extended from the well surface by their attachment through their peptide carboxyl-termini, on a molecular tether of average estimated length of 50 lysine subunits. This simply assumes that each polylysine molecule is attached to the surface through one bond at its midpoint. It is expected that multiple attachment bonds between the surface and a single polylysine chain can also form but would be minimized by the 2000-fold molar excess of polylysine over attachment capacity used during the polylysine coating step. Model experiments have shown that the polylysine helical structure would be maintained in solution [14,15] for most, if not all cases. If multiple attachment bonds were formed then distal ends would prefer the helical form while the sequences between attachment points should also prefer the helical form up to the limits imposed by torsional constraints, and depending on the closeness of the attachment positions. Thus, the peptides are well positioned spatially, to interact with a variety of macromolecules such as antibodies and structures as large as a cell surface. The lengths and sequences of the peptides are programmable; the total elapsed cycle time to extend each peptide by one residue for all 96 wells is 1 hour. The process has been optimized with respect to activation conditions, length and composition of the polymeric tether and conditions for the handling and storage of the pre-diluted amino acid derivatives and condensing agents. Stabilities exceeding two years have been achieved for all reagents used. The capability of these peptides to be recognized by antibodies, leading to the identification of sequences and structures of the immunoreactive domains of viral proteins and biological response modifiers has been previously been demonstrated [11-13].

### Reproducibility and quality of synthetic peptides

**Figure 2:**
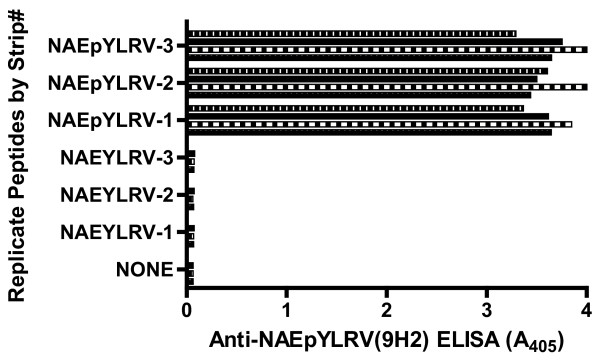
**Reproducibility and specificity of multiplexed EGFR peptide synthesis and ELISA testing **Peptides were synthesized as microtiter plate array strips using either tyrosine or phosphotyrosine. Three replicate strips were used, each with no peptide in row A and alternating phosphotyrosine and tyrosine peptides in rows B to H. The strips were tested for reactivity with clone 9H2 antibody as described in Methods. ELISA results (x-axis) are plotted against tyrosine or phosphotyrosine replicates for each strip (y-axis). Standard deviations varied between 5% and 10% of the means. Data are displayed without any correction.

To evaluate the fidelity and reproducibility of peptide syntheses and ability of the synthetic peptide arrays to serve as specific targets in sequence defined molecular affinity interactions, a model system was chosen to provide known test parameters. The EGF receptor system was selected since it provided commercially available monoclonal antibodies with documented specificity for an activation state associated autophosphorylation site (pY1173) and known sequence (NAEpYLRV). Testing was begun with the monoclonal antibody 9H2 produced against a peptide containing the NAEpYLRV sequence. An array of alternating NAEYLRV and NAE(pY)LRV peptides was prepared in a microtiter plate consisting of 12 8-well strips. Monoclonal 9H2 antibody ELISA was performed using three of the strips from the middle section of the plate. It was found that the antibody reacted strongly with the peptide wells containing phosphorylated tyrosine but not with the non-phosphorylated peptide wells or control wells without peptide (Figure 2). For the three strips ELISA means and (standard deviations) for the phosphotyrosine peptides were 3.62(0.20), 3.64(0.25), 3.67(030) and for the tyrosine peptides were 0.071(0.003), 0.068(0.007), 0.076(0.004). For wells containing no peptide, the values for all three strips were 0.063(0.0006). The coefficients of variation for all replicate sets ranged between 5% and 8%.

**Figure 3:**
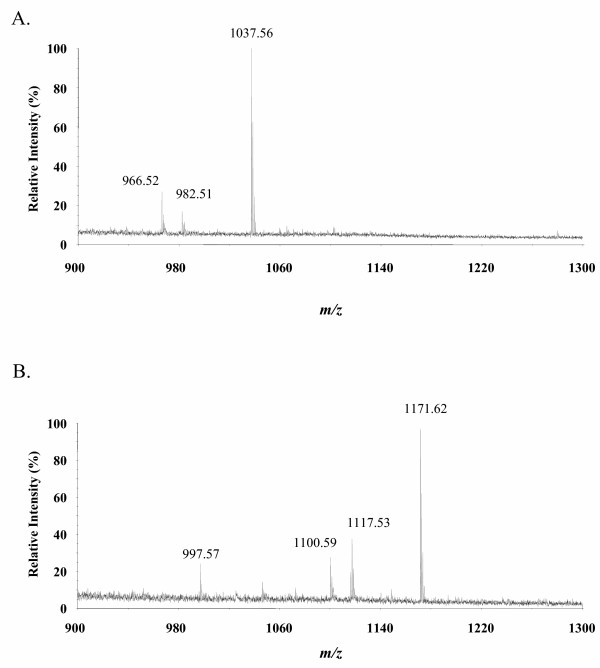
**Mass spectrometric characterization of EGFR phosphopeptides produced in microtiter plate arrays **Panel A illustrates the mass spectrum of the NAEYLRV derivative ([M+H]^1+ ^ion at *m/z *1037.56) and panel B for the NAEpYLRV derivative ([M+H]^1+ ^ion at *m/z *1170.62). Peptides were released by incubation for 3 hours in TFA-H_2_O-triisopropylsilane, concentrated *in vacuo *and reconstituted for MALDI-TOF-TOF analysis. Under these conditions the bis(dimethylamino) phosphate protecting groups of phosphotyrosine were preserved and mass spectra were improved.

For evaluation of the fidelity and authenticity of phosphorylated and non-phosphorylated peptide products wells containing NAEYLRV and NAEpYLRV were prepared as before except that a cleavable linker was added to the polylysine matrix before peptide synthesis. The synthesized peptides were then cleaved from the surface with TFA using conditions under which the protecting groups were removed from the amino acid side-chains but not from tyrosine phosphates. When the released peptide products were concentrated and analyzed by MALDI-TOF-TOF mass spectrometry it was found that both peptides yielded essentially monodisperse m/z of the predicted molecular weights, 1036.56 for NAEYLRV and 1170.62 for NAEpYLRV (Figure 3A and 3B, respectively; [bis(dimethylamino)phosphono]-tyrosine species shown). These data demonstrate high coupling efficiency at each step of synthesis and stability of the activated amino acids throughout the process.

### Redundancy of an autophosphorylation site antibody epitope in the EGFR cytoplasmic domain

**Figure 4:**
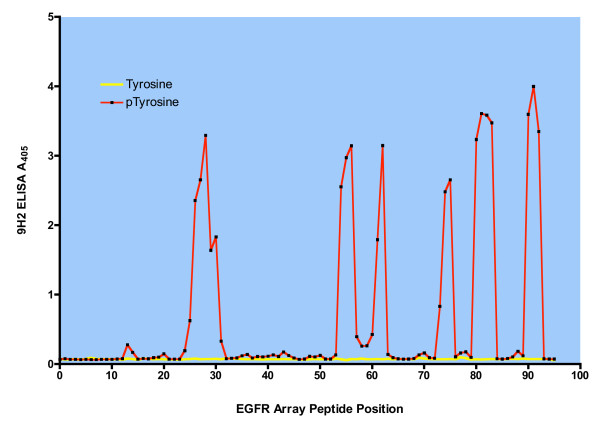
**Distribution of 9H2 phosphotyrosine epitopes in EGFR **Overlapping peptide arrays were made and acetylated in microtiter plates using human EGF receptor residues 622–1186 (Swissprot database locus P0053 with leader sequence removed). Each array contained exclusively tyrosine or phosphotyrosine peptides and was composed of 92 peptides each of which was 21 amino acids long and offset from its neighbor by 6 amino acids. Array peptide wells were reacted in multiplexed ELISA format with antibody 9H2 as described in Methods. Data are plotted without any corrections. Peptide sequences and raw data are listed [see Additional file 1].

Since many of the twenty tyrosines found in the EGFR cytoplasmic domain are known to serve as substrate or SH2 binding site for other tyrosine kinases, it was decided to test the specificity of the 9H2 antibody. To examine this question, overlapping peptide arrays covering the transmembrane and cytoplasmic domains of the EGF receptor were prepared. One array contained only phosphotyrosine and the other only tyrosine. The arrays consisted of 92 peptides, each of which was 21 amino acids in length and overlapped by 15 amino acids. In the EGFR sequence arrayed, there are 20 unique occurrences of tyrosine-containing peptide sequences. In the array, each such sequence appears three to four times in progressively overlapping fashion. Clone 9H2 antibody showed high reactivity (from 9 to 57 times background) with eight out of the twenty phosphotyrosine-containing sequences (Figure 4, also [see Additional file 1]). In general, each reactive peak was associated with three to four appearances of the identifiable tyrosine as its position moved progressively along the overlapping peptide sequences, suggesting consistent reliable synthesis throughout the synthetic process. None of the non-phosphorylated tyrosine-containing peptides showed any comparable reactivity with the monoclonal antibody 9H2 although a barely detectable level of antibody reactivity with all peptides containing tyrosine could be seen. There was no detectable reactivity against peptides not containing tyrosine [see Additional file 1]. Steric hindrance by the attachment matrix did not appear to be a significant problem in the recognition of reactive peptide sequences separated by just one, and two amino acids from the peptide carboxyl terminus ([see Additional file 1], array positions 81, 91)

**Figure 5:**
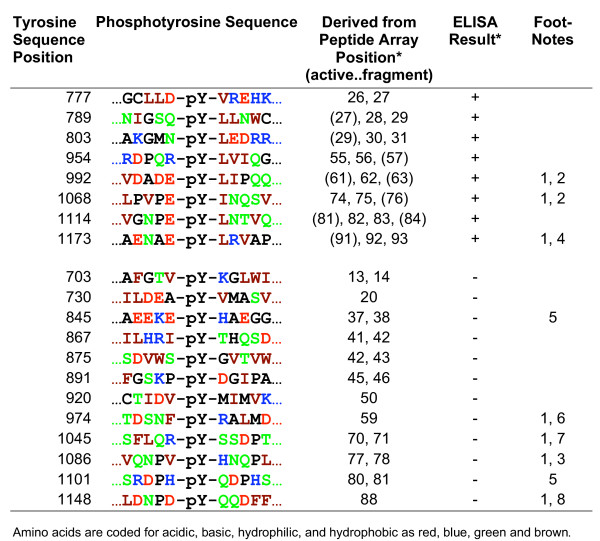
**Pattern of recognition of arrayed EGFR peptides by autophosphorylation site antibody (9H2) **Tyrosine sequence positions correspond to human EGF receptor residue positions (Swissprot P0053, leader sequence removed). Amino acids are color coded for acidic, basic, hydrophilic, and hydrophobic as red, blue, green and brown. *. For (phospho)peptide array compositions and quantitative results [see Additional file 1]. Footnotes: 1. autophosphorylation site [26-28], 2. PLC-gamma SH2 domain binding[29], 3. GRB2/SH2 domain binding [30], 4. Shc, SHP1, PLC-gamma SH2 domain binding [31, 32], 5. Src phosphorylation site [33-35],6. AP2 [36, 37], 7. Cbl, SH2 domain binding [38-40], 8. Shc SH2 domain binding [31, 32, 41].

To assess the significance of these primary cross-reactivities a consensus table was constructed from 9H2 antibody reactive and non-reactive sequences (Figure 5). A strong preference for hydrophobic amino acids in the Y+1 position is readily apparent with leucine the most preferred appearing in 6 of 8 peptides, followed by isoleucine and valine, both appearing once. At the Y-1 position glutamic acid was the most preferred, appearing in 4 of 8 peptides, followed by arginine, asparagine, aspartic acid, and glutamine with one appearance each. Thus the preference appears to be primarily for glutamic acid but other hydrophilic amino acids were also accepted. There did not appear to be any discernable pattern of preference at the remaining peptide positions. The least common denominator among positive peptides therefore appears to be E/(R, N, D, Q)-pY- L/(V, I), or E-pY-L in its predominant form. Peptide sequence # 84 [see Additional file 1] which lacked an acidic or hydrophilic amino acid at the pY-1 position was still strongly reactive demonstrating the strong contribution of a hydrophobic amino acid in the Y+1 position. However, since the peptide at position Y730 containing A(pY)V was not reactive ([see Additional file 1], Figure 5), 9H2 binding appears to involve more than the pY-hydrophobic sequence alone.

Three of the 9H2 cross-reactive phosphotyrosines are clustered within the C-terminal end of the receptor (992–1173), are known autophosphorylation sites and are known to be recognized by proteins containing Group III SH2 binding domains [16] (e.g. p85, phospholipase C_γ1_, the tyrosine phosphatases, Figure 5), that similarly recognize phosphotyrosines with hydrophobic amino acids at the Y+1 position. Since some of the 9H2 cross-reactive epitopes are not associated with phosphoacceptor activity it suggests that the phosphoacceptor site specificities are more stringently controlled than the 9H2 epitope or that phosphoacceptor activity simply has not been demonstrated yet. A similarity in the processes for recognition of specificity determinants within the deduced epitope of the 9H2 autophosphorylation site antibody and optimal substrate motif found for the EGF receptor [16-18], E**EEEYF**ELV, may also exist.

**Figure 6:**
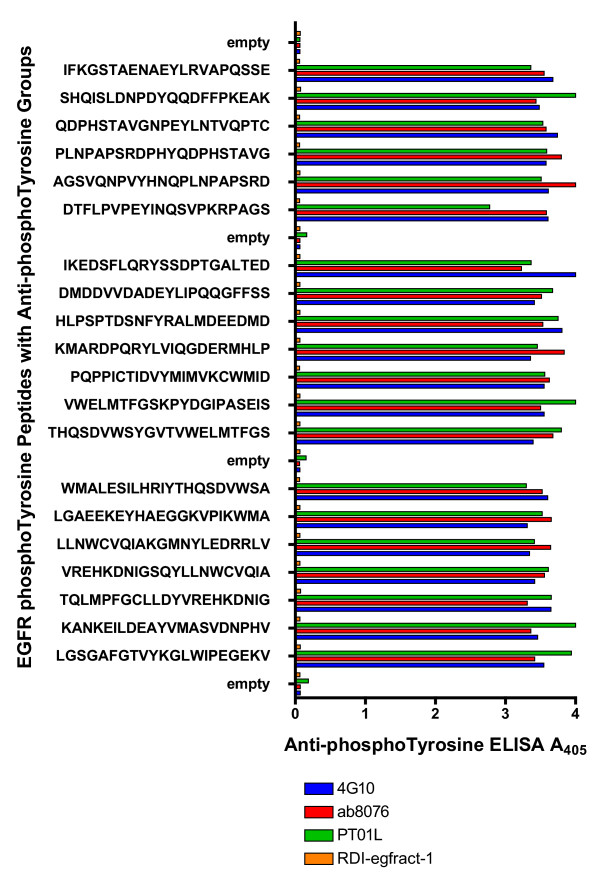
**Reactivity of all EGFR phosphotyrosine peptides with a panel of pan-specific phosphotyrosine monoclonal antibodies **Phosphotyrosine peptide sequences selected from the arrays used in Figure 4 [see Additional file 1] were synthesized in a new microtiter plate array and are identified on the y-axis. Each peptide and an empty control well were tested with each of the four antisera identified in the Figure legend, 4G10, ab8076, PT01L, and RDI-egfract-1 by standard ELISA as described in Methods. Data are plotted without any corrections

Twelve of the twenty tyrosines present in the EGFR cytoplasmic domain were not reactive with 9H2. None of the twelve conformed to the deduced 9H2-epitope motif, although six out of 12 were involved with other aspects of kinase signal transduction (Figure 5). As confirmation that this was not a result of failure to incorporate phosphotyrosine in the negative peptides a plate array containing all of the EGFR phosphotyrosine peptides was constructed and tested against a group of commercial phosphotyrosine antibodies prepared in various ways. These results are shown in Figure 6. Antibodies 4G10, AB8076, and PT101L were prepared using immunogens which were chemically modified or haptenized and not known to be sequence restricted and all reacted strongly with all of the EGFR phosphopeptides. Antibody RDI-egfract-1 was prepared against the activated EGF receptor and known to be activation specific but not known to be active against linear peptides. These results add support to the specificity of the epitope deduced above and contribute additional useful information and reagents, namely confirmation of the pan-specific nature of the phosphotyrosine antibodies as used in the microtiter plate peptide array system

### Tyrosine kinase activity on synthetic peptide substrates in microtiter plate arrays

**Figure 7:**
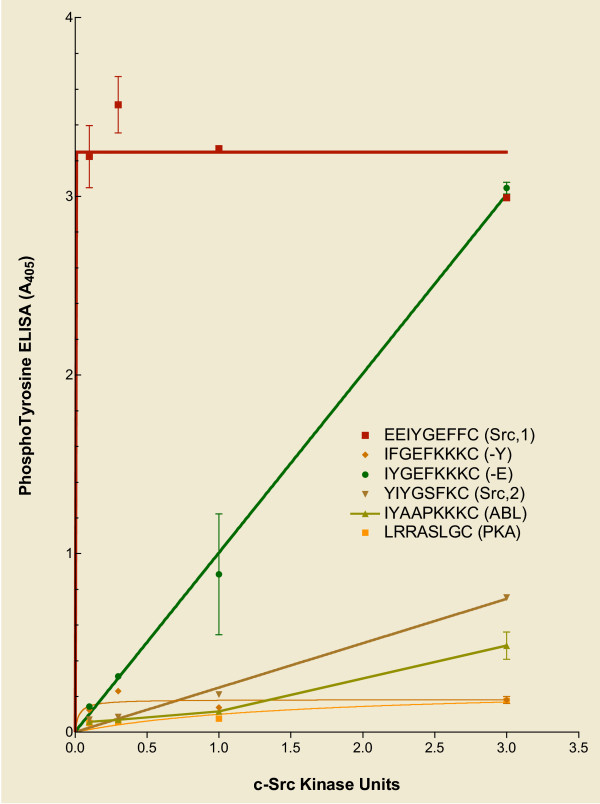
**Microtiter plate array peptides serve as substrates for p60^c-src ^with specificity and concentration dependence **Eight-well strips bearing six substrate peptide wells and two control wells were robotically synthesized *ab initio*, in microtiter plate format. All of the peptide substrates were initiated with Cys(Acm) to provide additional extension from the polylysine backbone and a common attachment site. The Cys-SH protecting group (Acm) remains in place and all peptides were acetylated at the amino terminus. Duplicate substrate strips were reacted with each 90-μL dilution of c-Src enzyme containing 3, 1, 0.3, and 0.1 Units of enzyme. The wells were reacted for 20' at 30°, washed with distilled water and assayed by antibody ELISA for the presence of phosphotyrosine using a mixture of antibodies described in Figure 5. Nonlinear regression plots were computed in Prism and error bars represent the means ± 1 SD, which ranged between 3% and 7% of mean values. Iterations for (ABL) did not converge. All data points were corrected by subtraction of values obtained from wells with no peptide.

To characterize the quantitative aspects of substrate phosphorylation by c-Src kinase, a panel of peptides based on known substrate specificities of c-src and related enzymes were synthesized in microtiter plate wells. Each peptide extended from the polylysine backbone by an ε-amino side-chain and by an additional C-terminal Cys unit. Thus, all of the peptides were equally available to the enzyme, with reduced steric hindrance and a uniform presentation. Subsequently, 90 μL of reaction buffer containing varying amounts of c-Src kinase were incubated for 20 min at 30°, according to the manufacturer's instructions. The wells were then washed with distilled water and assayed for the presence of phosphotyrosine by ELISA using a mixture of the broadly cross-reacting phosphotyrosine antibodies previously described in Figure 6. The peptide substrates **E**E**IYGEF**F [17] (Src,1) and YIYGSFK [19] (Src,2) have been shown separately by somewhat different combinatorial methods to have relatively potent activity for protein tyrosine kinases and are shown to be reactive here as well (Figure 7). (Src, 1) was phosphorylated to a greater extent than (Src, 2) and showed no decrease of reactivity even at 0.1 Unit of enzyme, the lowest concentration used. The (Src, 1) variant peptides were chosen so that validation could be made by direct comparison with the highly oriented peptide chip system recently described [20]. The (Src, 1) variant peptide (-E) made by truncation of the N-terminus still showed good reactivity, although much lower than the longer (Src,1) peptide, and the (-Y) peptide in which tyrosine was exchanged for phenylalanine showed no reactivity as expected and required. The slight c-ABL tyrosine kinase substrate peptide IYAAPKKK [17] reactivity at the highest c-Src input and negative protein kinase A substrate peptide LRRASLGC [21,22] activity are consistent with the level of cross-reactivities expected between familial and nonfamilial kinases. Three of the peptide substrates showed dose-response characteristics conforming, by nonlinear regression, to a one site binding model for (-E), (Src, 2), and (ABL), with R^2 ^equal to 0.98, 0.99, and 0.92. Activity of the most reactive peptide, (Src, 1) was greater than expected based on quantity of enzyme used in previously published work [23,24] and was at least 30 times more reactive than its truncated form (-E) [20]. By extrapolation, c-Src activity would be detectable at concentrations as low as 0.01 Units of enzyme using the (Src, 1) substrate.

## Conclusions

The method for production of synthetic peptide solid-phase arrays in microtiter plates described here is capable of making high quality peptides, as seen by mass spectrometry of the released unfractionated products. The peptide synthesis method is completely automated and has been greatly simplified by the use of standard automated liquid handlers and the use of activated amino acid solutions that may be prepared and stored in advance and added just once at the beginning of the synthetic process. In the present demonstration, 96 well microtiter plates were used; but, 384, 1536, or containers of any well density compatible with the solvents and liquid handler may be used. The solid phase peptide arrays produced on a polylysine backbone were found to be of very high density, provide very low levels of nonspecific binding and steric hindrance, and participate effectively in a variety of biochemical reactions.

The strategy described here for the preparation of solid phase synthetic peptide arrays in microtiter plate wells for use in multiplexed assays offers many advantages in the study of protein kinases, particularly in a research environment. In a research environment combined cycles of hypothesis generation and testing, with assay flexibility, speed, quantitative accuracy and precision are of greater concern than in large scale screening applications of large numbers using limited, previously selected variables. The industry standard microtiter plate format ensures compatibility with a vast number of assay platforms and the polylysine backbone with its extended three-dimensional display provides a highly efficient, sterically unhindered, and extremely low background display of the peptide products.

Using 96-member peptide arrays of 21-mers created in less than 24 hours, we have shown that the peptide array synthesis provided a highly reproducible model for a tyrosine peptide, EGFR Y1173 and its phosphorylated counterpart. Using a monoclonal antibody prepared against a synthetic peptide representation of the Y1173 EGF receptor autophosphorylation site, we have provided evidence that, unexpectedly, the deduced epitope, E/H_L_-pY-L/H_B _(where H_L_- is hydrophilic and H_B _is hydrophobic) is highly redundant within the cytoplasmic domain. Three of the eight antibody reactive sites have been previously identified as autophosphorylation sites and are recognized by Group III SH2 domain proteins (Figure 5) that have similar specificity patterns. Furthermore, the EGFR substrate sequence EEEEYFELV, derived by combinatorial peptide optimization [17], resembles the E-Y-(hydrophobic) motif found in these studies. Thus there is a consistent linkage between a subset of EGFR phosphotyrosine sequences recognized by the 9H2 antibody, a subset of sequences autophosphorylated by EGFR kinase, and EGFR autophosphorylation sites recognized by the Group III SH2 domain. There is a clear parallel between the 9H2 peptide epitope and the peptide substrate specificity of the EGFR catalytic activity [16]. Songyang has further suggested that the catalytic and SH2 domains of PTKs may have converged to recognize similar sequences. So, questions regarding which site (or sites) the antibody actually recognizes on stimulated EGF receptor molecules and what other parallels might exist between 9H2 binding, SH2 binding, and catalytic substrate selection become of interest. At the cell protein level, 9H2 is specific by Western blot for the stimulated EGF receptor. Furthermore, there are preliminary data (Saxinger, unpublished) suggesting that PDGF peptide sequences conforming to the 9H2 binding sequence of (hydrophilic)-pY-(hydrophobic) deduced from EGFR phosphotyrosine peptides, appear to be recognized differently by 9H2. While nine of the twenty-seven PDGFR phosphotyrosine sequences satisfied the (hydrophilic)-pY-(hydrophobic) sequence definition, and could be expected to be as reactive as those in EGFR, only one showed comparable reactivity. Thus, the binding determinants of 9H2 are more complex than the simple epitope deduced from Figure 5. An intriguing possibility is that the 9H2 epitope may be a fairly simple one but that its appearance, or access to it has been limited or distorted in specific ways by spatially adjacent sequences or structures that have evolved to create opportunities for exploitation in biologically specific processes.

We have also successfully demonstrated the use of microtiter plate peptide arrays in faithfully reproducing the known substrate phosphorylation specificities of c-Src protein kinase. In these studies a broadly reactive phosphotyrosine antibody ELISA detected phosphorylation. Although a mixture of antibodies that were not known to be sequence restricted was used, the possibility exists that some tyrosine-containing peptides could become phosphorylated and be recognized less well than others. Therefore in studies where the need for precise quantitation outweighs the convenience and safety considerations of ELISA, incorporation of radioisotopic phosphate would provide an alternative. The microtiter plate format with reactants bound to the well surface would provide a well-contained and safe vehicle for washing and subsequent measurement of radioactivity.

Thus, the microtiter plate array system is well suited to the study of protein kinase substrates, antigens, related binding molecules, and inhibitors since these all can be quantitatively studied at a single uniform, reproducible interface. For applications requiring larger numbers of solid phase peptides, the synthetic process can easily be transferred to more powerful workstations such as the Biomek FX platform in which many plates with higher well densities can be synthesized simultaneously and conventional particle based substrates for peptide synthesis can be manipulated using filter plates or magnetic devices available for this system. Moreover, the current capacity of approximately 150–380 pMoles/well can be considered large enough for preparative or analytical applications coupled with mass spectrometric analyses, such as affinity-based proteomic screening for ligand-protein interactions. In this and other applications, such as assessment of protease activity where strict isolation of adjacent components is required, microtiter plate wells enjoy a significant mechanical advantage over two-dimensional spotting or other synthesis methods.

## Methods

### Materials

Solvents and reagents for peptide synthesis were obtained as synthesizer grade from Applied Biosystems (Foster City, CA). Specifically, these were N-methylpyrollidone (NMP), 1 M N-Hydroxybenzotriazole (HOBT) in NMP, 1 M dicyclohexylcarbodiimide (DCC) in NMP, diisopropylethylamine (DIPEA), acetic anhydride, and trifluoroacetic acid (TFA). NMP from this vendor was consistently free of basic impurities and was stored over Molecular Sieve (4A) after opening. NMP solutions were stored at -20C and allowed to attain room temperature before opening. FMOC-protected α-amino acids were purchased from Penninsula Laboratories (San Carlos, CA) and side-chain substitutions were, Asn(Trt), Asp(OtBu), Cys(Acm) or Cys(Trt), Gln(Trt), Glu(OtBu), His(Trt), Lys(Boc), Ser(tBu), Thr(tBu) and Tyr(tBu). FMOC-Arg(Pbf), FMOC- [bis(dimethylamino)phosphono))-tyrosine] [25], and FMOC Rink amide (linker p- [(R,S)-a-[1-(9H-Fluoren-9-yl)-methoxyformamido]-2,4-dimethoxybenzyl]-phenoxyacetic acid) were purchased from NovaBiochem. Phenol, thioanisole, ethanedithiol (EDT), triisopropylsilane (TIPS), and carbonyldiimidazole (CDI) were obtained from Aldrich Chemical Co. (Milwaukee, WI). Poly(D-Lys•HBr) (dp 100) and Poly(L-Lys•HBr) (dp 100) were obtained from Sigma-Aldrich (St. Louis, MO). TPX (polymethylpentene) 8-well strips, non-sterile, non-tissue culture treated, were obtained from Costar on special order (Cambridge, MA).

### (Phospho)Peptide synthesis

Automated preparation of solid phase synthetic peptide arrays in microtiter plates was performed as described previously [13] (Saxinger, US Patent 6031074, Feb. 29, 2000). Carboxyl functional groups are formed on the hydrocarbon surface of Costar TPX microtiter plate strip wells by oxidation with 70% nitric acid for two hours at 65°C or for two weeks at room temperature. Polylysine chains are then attached to the surface by condensation using 0.05 M carbonyldiimidazole in NMP for 30' at 20°C. Poly(L-Lys•Hbr) (PLL) or poly(D-Lys•Hbr) (PDL) (1 mg/ml in 90% NMP-10% water) was neutralized with DIPEA and reacted with the CDI-activated surface for 1 hour at 20°C and followed by 4°C overnight. Plates were rinsed twice with water and twice with methanol, air-dried, wrapped in plastic cling-wrap and stored at room temperature. Peptide synthesis next takes place by the sequential addition of Nα-FMOC amino acids activated with DCC/HOBT as in conventional peptide synthesis with the growing peptide chain covalently attached to the polylysine chain through its carboxyl terminus. Stock solutions of FMOC amino acids were prepared in advance by dissolving 6 mmoles of each in 12 mL of NMP containing a 10% molar excess of HOBT, and stored at -20°C. The solutions could then be used repetitively for at least three months. Prior to peptide synthesis, the required amounts of amino acid were diluted to 0.1 M in NMP, mixed with an equimolar volume of 0.1 M DCC in NMP and allowed to react for thirty min in 2 mL cryovials. The 20 mixtures were then placed in a 24 tube rack on the Biomek 1000 workstation tablet for use during subsequent peptide synthesis cycles. Synthesis automation is achieved through software that receives input designating the peptide desired in each of the 96 microtiter plate wells and creates output files to indicate the quantity of reagents needed and a set of Beckman Biomek 1000 arrays.bio files, each member of the set directing the distribution of amino acids in one of the sequential chain extension cycles. An automated repetitive set of reagent transfers and washing steps is then recycled using a new arrays.bio file for each cyle until the peptide synthesis is complete. After synthesis the peptides are usually capped by reaction with acetic anhydride (1 mL in 10 mL of NMP containing 0.1 mL of DIPEA per plate) before side-chain deprotection and testing, in multiplex fashion, for reactivity. Side chain protecting groups were generally removed with: 10 mL of TFA +0.75 g of crystalline phenol + 0.5 mL of purified water + 0.5 mL of thioanisole +0.25 mL of ethanedithiol per plate, incubated for three hours in a sealed container, washed with ether, air-dried in a chemical fume hood, and stored at -20°C in plastic wraps. For peptides not containing Met, Trp, or Cys, TFA containing TIPS and H_2_O was used (95%, 2.5%, and 2.5%, respectively).

### Peptide synthesis capacity of microtiter plate wells

TPX microtiter plates were oxidized at either room temperature or 60°C and reacted with polylysine. Peptide quantities were estimated by two different methods. In the first, the polylysine amino groups were modified with the cleavable linker HMPB [4-(4-Hydroxymethyl-3-methoxyphenoxy)-butyric acid] and FMOC-L-Cys(Acm) was coupled to the HMPB-substituted support as described above. Terminal amino groups were deprotected with 20% piperidine in NMP, washed with NMP and derivatized with dabsyl chloride. The dabsyl-amino acid derivative was released with 95% TFA, harvested from multiple wells by serial transfer, and measured by spectrophotometric scanning in a Beckman DU65 using an extinction coefficient of Dabsyl Chloride = 6.5 × 10^4 ^in TFA, λ_max _= 495). In the second method, FMOC-L-Ala was coupled directly to the polylysine supports. The FMOC protecting group was released in 20% piperidine, harvested from multiple wells by serial transfer, and quantitated by fluorimetric scanning in a Perkin Elmer LS50B using E(301) = 7800 (1 mM/ml). Values for spectral constants and coefficients were determined from standards dissolved in the cleavage solvent. The solid phase capacity was 380 pMoles/well and the releasable capacity was 150 pMoles/well.

### Mass Spectrometry of synthetic array peptides

A polylysine coated TPX microtiter plate was modified by preliminary reaction with FMOC-Rink Amide linker (Novabiochem) to allow peptide cleavage after synthesis using standard DCC/HOBT coupling conditions, as described above. All peptides were initiated with C-terminal Cys(Acm) to provide a constant initiation and cleavage environment. Peptides were released and deprotected by incubation for 3 hours at room temperature in 100 μL of TFA containing water, ethanedithiol and phenol, or TFA containing water and triisopropylsilane. An additional 10 uL of water and 16 hours of incubation time are required for deprotection of [bis(dimethylamino)phosphono))-tyrosine] [25]. Solutions of released peptides were concentrated by vacuum centrifugation and 0.25 μL of sample was co-crystallized with 0.25 μL of α-cyano-4-hydroxycinnamic acid in 50% ACN, 1% trifluoroacetic acid and spotted directly on a stainless steel matrix-assisted laser desorption ionization (MALDI) plate. Mass spectra were acquired using an Applied Biosystems 4700 MALDI-TOF/TOF mass spectrometer (Applied Biosystems, Foster City, CA). MALDI mass spectra were externally calibrated (<20 ppm) using a standard peptide mixture.

### Antibody recognition of microtiter plate arrayed peptides

Goat anti-mouse IgG (phosphatase conjugated) was purchased from Kirkegaard & Perry (Gaithersburg, MD) and Upstate Biotechnology (Lake Placid, NY). Murine monoclonal antibody 9H2, purchased from Upstate Biotechnology was prepared against a synthetic peptide containing the EGFR autophosphorylation sequence at Y1173 and was certified by the manufacturer to be specific for EGF-stimulated A431 cells by Western blot. Murine antisera prepared against phosphotyrosine were 4G10 (Upstate), ab8076 (Abcam, Ltd.), and PT01L (Oncogene Research Products). Antiserum RDI-egfract-1 was prepared against isolated tyrosine phosphorylated (activated) EGF receptor from EGF-challenged murine L-cells (Research Diagnostics, Inc.). All sera were used according to the manufacturer's recommendations and assayed by indirect ELISA. The extent of reaction was determined using a phosphatase assay kit from Kirkegaard & Perry.

### Kinase assay

Peptide array substrate evaluations were performed using p60^c-src ^protein-tyrosine kinase, Cat# PK03 (Oncogene Research Products) according to the instructions in the manufacturer's insert. In sequence, all wells received 30 μL of Kinase Assay Buffer (0.05 M HEPES, pH 7.5 + 0.1 mM EDTA + 0.015% BRIJ 35), 30 μL of appropriately diluted p60^c-src ^in Kinase Dilution Buffer (0.1 mg/mL BSA + 0.2% β-mercaptoethanol), 30 μL of ATP mix (0.03 M MgCl_2 _+0.15 mM ATP) in Kinase Assay Buffer. Plates were incubated at 30°C for 30 min, rinsed with distilled water and assayed for the presence of phosphotyrosine as described above. Each unit of p60^c-src ^enzyme catalyzes the incorporation of one pMole of phosphate into tyrosyl residues.

## List of abbreviations used

(9H2): antibody prepared against a synthetic peptide containing the EGFR autophosphorylation site at Y1173

carbonyldiimidazole (CDI)

dicyclohexylcarbodiimide (DCC)

diisopropylethylamine (DIPEA)

EGF receptor (EGFR)

ethanedithiol (EDT),

N-Hydroxybenzotriazole (HOBT)

N-methylpyrollidone (NMP)

phosphotyrosine kinase (PTK)

Polymethylpentene (TPX)

trifluoroacetic acid (TFA)

triisopropylsilane (TIPS),

Amino acid and polypeptide abbreviations were in accordance with IUPAC-IUB recommendations.

## Authors' contributions

WCS designed the strategy for peptide synthesis, participated in the design of the strategy for peptide synthesis validation, carried out the peptide synthesis, testing, functional analyses, and drafted the manuscript. TPC participated in the design of the strategy for peptide synthesis validation, carried out the mass spectrometry analyses and interpreted the ms results. DJG participated in the design of the strategy for peptide synthesis validation. TDV participated in the design of the strategy for peptide synthesis validation and coordinated the mass spectrometry analyses.

## Supplementary Material

Additional File 1 EGRF (pY) peptide scanning array Vs 9H2 Antibody ELISA. EGF receptor phosphotyrosine and tyrosine overlapping peptide array sequences and ELISA test results for each peptide array. Click here for file
