# Supplementary material for: Fully automated synthesis of (phospho)peptide arrays in microtiter plate wells provides efficient access to protein tyrosine kinase characterization
Source: BMC Immunol. 2005 Jan 12;6:1. doi: 10.1186/1471-2172-6-1 (PMC546003; doi:10.1186/1471-2172-6-1)
Supplement: Additional File 1 — EGRF (pY) peptide scanning array Vs 9H2 Antibody ELISA. EGF receptor phosphotyrosine and tyrosine overlapping peptide array sequences and ELISA test results for each peptide array. [file 1471-2172-6-1-S1.pdf]

9H2 Antibody Responses to EGFR Array of Overlapping phosphotyrosine Peptides Sorted by Sequence #

Array Well Position #	EGFR phosphotyrosine Peptide*	9H2 Antibody ELISA	Relation to Deduced Epitope
1	empty (control)	0.068	
2	IATGMVGALLLLLVVALGIGL	0.078	
3	GALLLLLVVALGIGLFMRRRH	0.068	
4	LVVALGIGLFMRRRHIVRKRT	0.067	
5	IGLFMRRRHIVRKRTLRRLLQ	0.065	
6	RRHIVRKRTLRRLLQERELVE	0.068	
7	KRTLRRLLQERELVEPLTPSG	0.064	
8	LLQERELVEPLTPSGEAPNQA	0.064	
9	LVEPLTPSGEAPNQALLRILK	0.068	
10	PSGEAPNQALLRILKETEFKK	0.068	
11	NQALLRILKETEFKKIKVLGS	0.068	
12	ILKETEFKKIKVLGSGAFGTV	0.074	
13	FKKIKVLGSGAFGTVYKGLWI	0.078	
14	LGSGAFGTVYKGLWIPEGEKV	0.278	
15	GTVYKGLWIPEGEKVKIPVAI	0.169	
16	LWIPEGEKVKIPVAIKELREA	0.071	
17	EKVKIPVAIKELREATSPKAN	0.079	
18	VAIKELREATSPKANKEILDE	0.075	
19	REATSPKANKEILDEAYVMAS	0.094	
20	KANKEILDEAYVMASVDNPHV	0.099	
21	LDEAYVMASVDNPHVCRLLGI	0.148	
22	MASVDNPHVCRLLGICLTSTV	0.07	
23	PHVCRLLGICLTSTVQLITQL	0.071	
24	LGICLTSTVQLITQLMPFGCL	0.07	
25	STVQLITQLMPFGCLLDYVRE	0.194	homologous
26	TQLMPFGCLLDYVREHKDNIG	0.627	homologous
27	GCLLDYVREHKDNIGSQYLLN	2.357	overlap
28	VREHKDNIGSQYLLNWCVQIA	2.653	partial
29	NIGSQYLLNWCVQIAKGMNYL	3.295	overlap
30	LLNWCVQIAKGMNYLEDRRLV	1.639	partial
31	QIAKGMNYLEDRRLVHRDLAA	1.831	partial
32	NYLEDRRLVHRDLAARNVLVK	0.331	partial
33	RLVHRDLAARNVLVKTPQHVK	0.077	

34	LAARNVLVKTPQHVKITDFGL	0.083	
35	LVKTPQHVKITDFGLAKLLGA	0.091	
36	HVKITDFGLAKLLGAEEKEYH	0.121	
37	FGLAKLLGAEEKEYHAEGGKV	0.139	
38	LGAEEKEYHAEGGKVPIKWMA	0.089	
39	EYHAEGGKVPIKWMALESILH	0.11	
40	GKVPIKWMALESILHRIYTHQ	0.105	
41	WMALESILHRIYTHQSDVWSY	0.113	
42	ILHRIYTHQSDVWSYGVTVWE	0.133	
43	THQSDVWSYGVTVWELMTFGS	0.109	
44	WSYGVTVWELMTFGSKPYDGI	0.174	
45	VWELMTFGSKPYDGIPASEIS	0.123	
46	FGSKPYDGIPASEISSILEKG	0.088	
47	DGIPASEISSILEKGERLPQP	0.069	
48	EISSILEKGERLPQPPICTID	0.073	
49	EKGERLPQPPICTIDVYMIMV	0.112	
50	PQPPICTIDVYMIMVKCWMID	0.105	
51	TIDVYMIMVKCWMIDADSRPK	0.126	
52	IMVKCWMIDADSRPKFRELII	0.074	
53	MIDADSRPKFRELIIEFSKMA	0.073	
54	RPKFRELIIEFSKMARDPQRY	0.133	
55	LIIEFSKMARDPQRYLVIQGD	2.552	partial
56	KMARDPQRYLVIQGDERMHLP	2.974	partial
57	QRYLVIQGDERMHLPSPTDSN	3.145	partial
58	QGDERMHLPSPTDSNFYRALM	0.395	
59	HLPSPTDSNFYRALMDEEDMD	0.257	
60	DSNFYRALMDEEDMDDVVDAD	0.263	
61	ALMDEEDMDDVVDADEYLIPQ	0.426	Identical
62	DMDDVVDADEYLIPQQGFFSS	1.791	Identical
63	DADEYLIPQQGFFSSPSTSRT	3.147	Identical
64	IPQQGFFSSPSTSRTPLLSSL	0.138	
65	FSSPSTSRTPLLSSLSATSNN	0.094	
66	SRTPLLSSLSATSNNSTVACI	0.075	
67	SSLSATSNNSTVACIDRNGLQ	0.07	
68	SNNSTVACIDRNGLQSCPIKE	0.074	
69	ACIDRNGLQSCPIKEDSFLQR	0.079	
70	GLQSCPIKEDSFLQRYSSDPT	0.133	
71	IKEDSFLQRYSSDPTGALTED	0.161	
72	LQRYSSDPTGALTEDSIDDTF	0.092	

73	DPTGALTEDSIDDTFLPVPEY	0.084	
74	TEDSIDDTFLPVPEYINQSVP	0.833	homologous
75	DTFLPVPEYINQSVPKRPAGS	2.481	homologous
76	PEYINQSVPKRPAGSVQNPVY	2.653	homologous
77	SVPKRPAGSVQNPVYHNQPLN	0.106	
78	AGSVQNPVYHNQPLNPAPSRD	0.161	
79	PVYHNQPLNPAPSRDPHYQDP	0.175	
80	PLNPAPSRDPHYQDPHSTAVG	0.096	
81	SRDPHYQDPHSTAVGNPEYLN	3.236	overlap
82	QDPHSTAVGNPEYLNTVQPTC	3.609	Identical
83	AVGNPEYLNTVQPTCVNSTFD	3.585	Identical
84	YLNTVQPTCVNSTFDSPAHWA	3.475	Identical
85	PTCVNSTFDSPAHWAQKGSHQ	0.077	
86	TFDSPAHWAQKGSHQISLDNP	0.072	
87	HWAQKGSHQISLDNPDYQQDF	0.08	
88	SHQISLDNPDYQQDFFPKEAK	0.102	
89	DNPDYQQDFFPKEAKPNGIFK	0.184	
90	QDFFPKEAKPNGIFKGSTAEN	0.12	
91	EAKPNGIFKGSTAENAEYLRV	3.599	Identical
92	IFKGSTAENAEYLRVAPQSSE	4	Identical
93	AENAEYLRVAPQSSEFIGA	3.35	Identical
94	empty (control)	0.075	
95	empty (control)	0.073	
96	empty (control)	0.072	

*EGFR residues 646-1210, Length=21, Offset=6.
